# Developing a guideline for measuring workplace non-Gaussian noise exposure based on kurtosis adjustment of noise level in China

**DOI:** 10.3389/fpubh.2022.1003203

**Published:** 2022-09-23

**Authors:** Meibian Zhang, Yong Hu, Wei Qiu, Xiangjing Gao, Anke Zeng, Zhihao Shi, Jiarui Xin, Shixing Bai, Xin Sun

**Affiliations:** ^1^Chinese Center for Disease Control and Prevention, National Institute of Occupational Health and Poison Control, Beijing, China; ^2^Zhejiang Provincial Center for Disease Control and Prevention, Occupational Health and Radiation Protection Institute, Hangzhou, China; ^3^Auditory Research Laboratory, State University of New York at Plattsburgh, Plattsburgh, NY, United States; ^4^Jiaxing Center for Disease Control and Prevention, Jiaxing, China; ^5^Yangtze Delta Region Institute of Tsinghua University, Jiaxing, China

**Keywords:** non-Gaussian noise, complex noise, measurement, kurtosis, guideline

## Abstract

**Objective:**

There is no unified standard for measuring workplace non-Gaussian noise (known as complex noise) exposure. This study aimed to develop a draft guideline for measuring workplace non-Gaussian complex noise exposure based on noise temporal structure adjustment.

**Methods:**

Noise exposure level, e.g., the A-weighted sound pressure level normalized to a nominal 8-h working day (L_EX,8h_), was adjusted using the temporal structure (expressed by kurtosis) of noise. Noise waveform analysis or the instrument's direct reading was used.

**Results:**

The framework of the draft guideline included measurement metrics, the protocol using kurtosis to adjust L_EX,8h_, technical requirements for measuring instruments, measurement steps, data analysis, and measurement recording.

**Conclusion:**

The draft guideline could provide a basis for accurately measuring workers' exposure to non-Gaussian noise.

## Introduction

Noise is one of the most common occupational hazardous factors in the occupational environment. Noise-induced hearing loss (NIHL), a type of progressive sensorineural hearing loss, has become a global public health problem. World Health Organization (WHO) estimated that 10% of the global population is exposed to noise pollution, and of those, 6.2% suffer from NIHL (1). Approximately 16% of adult hearing loss was related to occupational noise (2). The prevalence of occupational NIHL was estimated at 10% in developed countries and 18–67% in developing countries, respectively (3). In China, occupational noise-induced deafness has become the second primary occupational disease after pneumoconiosis, with the number of reported cases increasing at an annual rate of 18.68% from 2010 to 2019 (4, 5).

Regarding its temporal structure, industrial noise can be divided into steady-state, continuous (Gaussian) noise, and non-Gaussian complex noise. Complex noise is composed of transient high-energy impulsive noise superimposed on Gaussian background noise, which is common in the occupational environment (6, 7). Existing noise exposure measurement standards (8) are suitable for steady-state noise to assess the noise exposure level. Because the complex noise contains impulsive components, it is challenging to measure the complex noise in industrial settings. Conventional noise measurement techniques, which only use the noise energy metric of sound pressure level (L_eq_), are not suitable for complex noise measurement due to the peak clipping effect of impulse noise (9). Moreover, these conventional noise measurement instruments (e.g., dosimeters or sound level meters) cannot measure the temporal structure of the noise.

The existing noise measurement standard is based on the “equal energy theory (EEH).” The principle of “EEH” is that the hearing damage caused by noise is only proportional to the noise energy; that is, no matter what type of noise, as long as the energy of noise is equal, it should cause the same damage to the hearing. A large number of animal experiments (10, 11) and epidemiological investigations (−19) have proved that exposure to complex noise leads to more severe hearing damage than steady noise, indicating that the “EEH” may underestimate the hearing loss induced by non-Gaussian complex noise. The problem with the existing standards is that the temporal structure of non-Gaussian noise is not considered in assessing the impact on hearing and only uses noise energy to characterize noise exposure. Therefore, it cannot fully reflect the hearing loss caused by complex noise. The measurement and evaluation of complex noise exposure should be combined with the energy metric of noise and the temporal structure metric, that is, the use of kurtosis to adjust the noise energy introduced in this paper.

Kurtosis is an indirect metric reflecting noise temporal structure (12). Animal experiments (10, 11) and epidemiological studies (12–19) suggest that kurtosis, a significant risk factor for NIHL, can adjust the noise energy metric into a combined metric. These kurtosis-adjusted noise energy metrics can accurately measure complex noise exposure and effectively assess the hearing loss caused by non-Gaussian noise. Although the measurement method of non-Gaussian noise based on kurtosis adjustment has been defined in human studies (12, 17, 18), there is no comprehensive measurement guideline for non-Gaussian noise exposure.

Based on our previous studies and literature reviews, we propose a draft guideline for China to measure workplace non-Gaussian complex noise exposure using kurtosis adjustment of the noise level. This draft guideline will stipulate a measurement standard and technical benchmarks for measuring non-Gaussian exposure in the workplace and provide a basis for protecting the hearing of noise-exposed workers, including vulnerable groups such as the elderly and women.

## Methods

### Terms and definitions

Relevant terms and their definitions in this guideline are shown in Table 1.

**Table 1:** Relevant terms and their definitions.

**Terms**	**Definitions**
Steady-state noise	Noise with sound level fluctuation <3dBA, also known as Gaussian noise
Non-Gaussian noise	The noise is composed of transient high-energy impulsive noise superimposed on Gaussian background noise, including classic impulsive noise, also known as complex noise
Kurtosis (β)	The ratio of the fourth-order central moment to the squared second-order central moment of a distribution, defines how heavily the tails of a distribution differ from the tails of a Gaussian distribution
Nominal day	Working day over which it is chosen to determine the noise exposure
Daily noise exposure level (L_EX,8h_)	A-weighted sound pressure level normalized to a nominal 8-h working day
Week noise exposure level (L_EX,40h_)	A daily noise exposure level normalized to a nominal week of five 8h working days
Task	A distinct part of workers' occupational activity
Job	Overall occupational activity that is carried out by a worker, consisting of all the tasks performed by the worker during the entire working day or shift
Homogeneous noise exposure group	A group of workers that are performing the same job and are expected to have similar noise exposure during a working day

### Measurement metrics

#### Noise energy metrics

The energy metric in existing noise measurement and evaluation criteria is the noise intensity, usually expressed by the A-weighted sound pressure level normalized to a nominal 8-h working day (L_EX,8h_).

L_EX,8h_ can be calculated by the formula (8):


(1) $$\begin{array}{l} LEX,8h=LAeq,Te+10*lg\left(Te/T0\right) \end{array}$$

where T_e_ is the effective duration of the working day in hours; T_0_ is the reference duration (8 h); L_Aeq, Te_ is the L_Aeq_ for T_e_.

If a normalized exposure level over a week is desired, it can be calculated as follows (20):


(2) $$\begin{array}{l} L_{EX40h}=10\;*\;\mathrm{lg}\left[\frac{1}{5}\sum_{i=1}^{5}\left(10^{0.1^{*}{\left(L_{EX,8h}\right)}_{i}}\right)\right] \end{array}$$

L_EX,40h_ is L_EX,8h_ normalized to a nominal week of five 8 h working days.

#### Noise temporal structure metrics

For a complex noise, noise temporal structure variables affecting hearing include the peak value, peak duration, and inter-peak distribution. In the actual measurement activity, it is not feasible to quantitatively analyze each of these variables to characterize the temporal structure for a shift-long noise exposure. Kurtosis, incorporating these time-domain variables into a simple metric, can quantify the impulsiveness of complex noise and is much more practical as a specific metric for the temporal structure of complex noise (12, 21). Kurtosis is a statistic that describes the normal distribution of probability distributions of random variables. The calculation formula is following:


(3) $$\begin{array}{l} \beta =\frac{\frac{1}{n}\sum_{i=1}^{n}{\left(xi-\bar{x}\right)}^{4}}{{\left[\frac{1}{n}\sum_{i=1}^{n}{\left(xi-\text{x}\right)}^{2}\right]}^{2}} \end{array}$$

Where β is the kurtosis; *x*_*i*_ is the i^th^ value of noise amplitude, and $\bar{x}$ is the sample mean. Kurtosis is a statistical measure of extreme values (or outliers) in data in either tail relative to a Gaussian distribution. Therefore, kurtosis is sensitive to the number of outliers. Put another way, kurtosis describes the tendency for a sound to have high amplitude events that depart substantially from the underlying, continuous, steady-state noise. Kurtosis can differentiate the degree of hearing loss caused by noise with different temporal structures at the same noise exposure level.

Kurtosis has some disadvantages: e.g., the length of intervals over which kurtosis is determined can affect the outcome and kurtosis has high sampling variability. Therefore, noise exposures can be clearly and effectively characterized by kurtosis only when the window length and the noise sampling rate are fixed or standardized. Based on the results of previous studies (17, 18, 22), the following protocol was established for kurtosis application in the evaluation of NIHL in industrial settings: The kurtosis of the recorded noise signal is computed over consecutive 60-s time windows without overlap over the shift-long noise record or whole measurement duration using a sampling rate of 48 kHz for noise recording. The mean of the measured kurtosis values, β_*j*_, at every 60 s is calculated and used as the kurtosis of noise exposure (β_*N*_):


(4) $$\begin{array}{l} \beta N=\frac{1}{N}\sum_{j=1}^{N}\beta j \end{array}$$

Theoretically, the kurtosis value of steady-state (Gaussian) noise is 3. However, the steady-state noise environment is rare in industrial production, and the quasi-Gaussian noise environment (kurtosis ranging from 3 to 10) often occurs with its energy distribution close to a normal distribution (Gaussian distribution).

### The adjustment protocol of noise level using kurtosis

The adjustment protocol applies kurtosis to adjust the noise intensity based on Goley's protocol from animal data (23). The calculation formula is as follows:


(5) $$\begin{array}{l} L_{EX,8h}-\text{K}=L_{EX,8h}+\lambda \;*\;\mathrm{lg}\left(\beta_{N}/3\right) \end{array}$$

In the formula, β is the kurtosis value of the noise measured; L_EX,8h_-K is kurtosis-adjusted L_EX,8h_; λ is the adjustment coefficient obtained from the dose-effect relationship between noise exposure and human hearing loss among a large sample of populations. In Goley's study, the λ value is 4.02 based on animal data. According to the multiple linear regression results from human data (18), the λ value is 6.5. The L_Aeq,8h_-K can be calculated as follows:


(6) $$\begin{array}{l} L_{EX,8h}-\text{K}=L_{EX,8h}+6.5\;*\;lg\left(\beta_{N}/3\right) \end{array}$$

where β_N_ is the average kurtosis value of noise during measurement duration. For example, when the average kurtosis of the noise is 30, the L_EX,8h_ increases by 6.5 dB(A) according to Formula 6. The calculation of L_EX,40h_-K can be performed according to Formula 6.

### Requirement of measuring instruments

L_Aeq_ measurement can follow an occupational health standard in China, i.e., “Measurement of Physical Agents in the workplace-Part 8: noise (GBZ/T 189.8),” which is based on the ISO 9612 (2009) “Acoustics-Determination of occupational noise exposure-engineering method.” The L_Aeq_ measurement can be made using either integrating-averaging sound level meters or personal sound exposure meters. The two kinds of instruments shall meet the requirements of IEC 61672-1:2002.

Measurement of kurtosis (β) needs a dedicated personal sound exposure meter (or noise dosimeter) with a recording or direct reading function. This kind of personal sound exposure meter can also measure the L_Aeq_ and L_EX,8h._ The digital instrument has at least one of the following functions: (1) sound recording for further analysis of kurtosis or L_Aeq_; (2) Automatic calculation of kurtosis, L_EX,8H_, or L_EX,8h_-K for direct reading. The primary technical requirements for the dosimeter are as follows (12–19): (1) a ¼” pre-polarized condenser microphone with a broad response frequency (20 Hz to 20 kHz) and high sensitivity level (2.24 mV/Pa); (2) The L_Aeq_ measurement ranges from 40 to 141 dB(A); (3) can work continuously for at least 16 h under full charge; (4) has an at least 32-GB memory card inside; (5) can record the noise continuously with a 32-bit resolution and at a sampling rate of 48 kHz.

## Results

### Measurement procedures

#### Field investigation

A field investigation is needed before noise measurement, which shall be carried out under normal production conditions and cover all workplaces involving noise exposure. The investigation includes the followings: (1) General information of enterprise, product, production process, and its zoning; (2) Source of noise, layout of noisy equipment, significant noise exposure event; (3) Work analysis. The field investigation aims to: (a) identify noise-related jobs; (b) define homogeneous noise exposure groups based on job title, function, and work area; (c) identify tasks that make up each job.

In the work analysis, workers' working-day recording of each noise-related job is needed as the original recording material in the noise measurement process. The contents shall include as follows: job title, the number of exposed workers, noise type, exposure time, exposure frequency, work shift, work sites and their change during the working day, task description, significant noise exposure events, and use of hearing protection devices. One or two representative workers per job or homogeneous noise exposure group are selected for the recording.

After finishing the field investigation, a noise measurement plan must be established, and a schematic diagram set with field sampling/measuring point must be drawn.

#### Preparation of instruments

Before measurement, the personal sound exposure dosimeter or sound level meter shall be fully charged, and a wind-proof cap of the microphone is prepared for use in a place where the wind speed is >3 m/s. Calibration of the instruments, including filed calibration, should be finished according to relevant calibration requirements.

#### Determination of sampling subjects

Kurtosis and L_EX,8h_-K are obtained from personal noise recording. Each subject shall confirm that this is the noise they are typically exposed to on an average working day.

The number of subjects for sampling is determined based on an occupational health standard in China (i.e., Specifications of air sampling for hazardous substance monitoring in the workplace): if the number of each job (or homogeneous noise exposure group) is <3 workers, all workers are selected; if the number of each job is 3–5 workers, two workers shall be selected; if the number of each job is 6–10, three workers shall be selected; if the number of each job is >10, four workers shall be selected.

#### Measurement of noise

Suppose the noise is determined as steady-state noise through the field investigation, the measurement for steady-state noise follows the conventional noise measurement standard, the “Measurement of Physical Agents in the workplace-Part 8: noise (GBZ/T 189.8)” in China, which is based on the “Acoustics-Determination of occupational noise exposure-engineering method (20).”

### Dosimeter wearing

Non-Gaussian noise measurement requires an appropriate way to wear the dosimeter. The dosimeter is clipped to the worker's collar, then mounted on the top of the shoulder at a distance of at least 0.1 m from the entrance of the external ear canal at the side of the most exposed ear and should be ~0.04 m above the shoulder. When recording, the microphone stands up to avoid the mechanical influence or clothing covering. The participants are trained to wear the dosimeter properly. The microphone should wear a wind-proof cap if the wind speed exceeds 3 m/s. The interference from electromagnetic fields shall be avoided as far as possible.

### Measurement methods

Measuring non-Gaussian noise has two methods based on the instrument's availability, i.e., noise waveform analysis and direct reading of the instrument.

Noise waveform analysis uses the specific noise dosimeter to record sound waves. Whole-shift sampling is preferred for recording the noise waveform. In order to facilitate practical operation, a long-time sampling with at least 1 h can be selected. The sampling period shall represent the whole-shift activity of each job, covering typical tasks that produce the cyclic fluctuating or randomly fluctuating noise. The recording is transferred to a computer for subsequent analysis. The noise waveforms are analyzed using a particular software (e.g., MATLAB) to calculate L_EX,8h_ and kurtosis value; Then, the L_EX,8h_-K is calculated.

The direct reading method uses the specific noise dosimeter with the automatic calculation of kurtosis and L_EX,8h_-K. The placement and measuring steps of the instrument is the same as the method of “Noise waveform analysis.” The kurtosis, L_EX,8h_, and L_EX,8h_-K values obtained from whole-shift measurement or long-time sampling with at least 1 h can be read directly and downloaded to the computer.

### Data analysis

Conduct the exposure assessment of non-Gaussian noise in workplaces based on a comparison between the L_EX,8h_-K and the occupational exposure limit for noise (i.e., 85dB(A)); Prioritize the noise impulsiveness among different jobs or tasks by comparing kurtosis levels of various jobs or tasks.

### Measurement records

Measurement records shall include the following: Measurement date, time, weather conditions (temperature, relative humidity, wind speed), field calibration of dosimeter, dosimeter, measurement location, job or task, significant noise exposure event, measuring duration, noise data, calculation formula and process, and signature of surveyor and accompanying personnel from the enterprise.

### Notes of non-Gaussian noise measurement

Because kurtosis is an adjunct metric to energy, it has been shown that kurtosis had an impact on NIHL evaluation only when L_EX,8h_ is ≥70 dB(A) (18). Therefore, the condition of using kurtosis adjustment (Equation 6) in assessment of NIHL is that L_EX,8h_ ≥70 dB (A). On the other hand, the optimal application range of Equation 6 is L_EX,8h_ between 70 and 95 dB (A), because Equation 6 was based on worker data at this noise level range. Workers exposed to this range of noise levels were not using hearing protection device at the time of data collection, so the data provide a reliable dose-response relationship. For L_EX,8h_ higher than 95 dB (A), Equation 6 provided a reasonable interpolation (18).

Measurement personnel shall pay attention to their hearing protection during the filed measurement and investigation. Measuring the C-weighted equivalent sound pressure level is recommended to select effective hearing protectors for measurement personnel and noise-exposed workers based on a standard in China (i.e. Guideline for selection of hearing protectors, GB/T 23466).

## Discussion

Studies have validated the adjustment methods for noise energy using kurtosis (12, 14, 15, 17, 19). Based on the definition of cumulative noise exposure (CNE) containing exposure duration and exposure level, there are two adjustment protocols; one is to adjust the exposure duration in CNE, and another is to adjust the noise exposure level, L_EX,8h_ or L_EX,40h_. This guideline does not include the adjustment of CNE. Although CNE is related to the prevalence of occupational hearing loss, its relationship with a specific degree of hearing loss, such as noise-induced permanent threshold shift (NIPTS), is unclear. In addition, the exposure duration (T) in CNE is calculated by years, and it is difficult to accurately investigate the exposure duration due to the frequent changes in workers' jobs and tasks.

This draft guideline must address several critical issues based on previous studies and literature reviews. (1) The validity of kurtosis to reflect noise's temporal structure: evidence shows that kurtosis can quantify the impulsiveness of complex noise and is much more practical as a specific metric for the temporal structure of complex noise (12, 21); (2) The validity of the adjustment protocol applies kurtosis to adjust the noise intensity: Formula 6 regarding the calculation of L_EX,8h_-K is developed based on our human study results (18) and Goley's protocol from animal data (23). The λ value of 4.02 proposed by Goley is adjusted to 6.5, obtained from the multiple linear regression results from human data. After the adjustment of L_EX,8h_ by kurtosis, we found that the underestimation of NIPTS346 by ISO 1999 improved. (3) The basis of the measurement procedures for non-Gaussian noise. The measurement procedures regarding field investigation, determination of sampling subjects, and dosimeter wearing were developed based on the “Measurement of Physical Agents in the workplace-Part 8: noise (GBZ/T 189.8)” in China, which is based on the “Acoustics-Determination of occupational noise exposure-engineering method (20).” In addition, measurement methods of non-Gaussian noise using individual sampling were developed based on our previous studies (12–19). The direct reading method of kurtosis and L_EX,8h_-K values is the preferred method in the future if the dosimeter with kurtosis function becomes commercially available. The prototype of the dosimeter with kurtosis function has been developed successfully in China.

## Data availability statement

The original contributions presented in the study are included in the article/supplementary material, further inquiries can be directed to the corresponding author/s.

## Author contributions

MZ: conceptualization, funding acquisition, and writing–original draft. YH: methodology and visualization. WQ: methodology. XG: funding acquisition. AZ, ZS, and JX: literature retrieval. SB and XS: conceptualization, writing–review & editing, and supervision. All authors contributed to manuscript revision, read, and approved the submitted version.

## Funding

This research was funded by the Zhejiang Provincial Key Research and Development Project (Grant Number: 2015C03039), the Zhejiang Provincial Program for the Cultivation of High-Level Innovative Health Talents, Zhejiang Province, China, the pre-research project on occupational health standards (20210102), and the Health Commission of Zhejiang Province (Grant Numbers: 2019KY057 and 2021KY120).

## Conflict of interest

The authors declare that the research was conducted in the absence of any commercial or financial relationships that could be construed as a potential conflict of interest.

## Publisher's note

All claims expressed in this article are solely those of the authors and do not necessarily represent those of their affiliated organizations, or those of the publisher, the editors and the reviewers. Any product that may be evaluated in this article, or claim that may be made by its manufacturer, is not guaranteed or endorsed by the publisher.
